# The potential therapeutic role of lymph node resection in epithelial ovarian cancer: a study of 13 918 patients

**DOI:** 10.1038/sj.bjc.6603803

**Published:** 2007-05-22

**Authors:** J K Chan, R Urban, J M Hu, J Y Shin, A Husain, N N Teng, J S Berek, K Osann, D S Kapp

**Affiliations:** 1 Division of Gynecologic Oncology, Department of Obstetrics, Gynecology, and Reproductive Sciences, University of California, San Francisco School of Medicine, University of California, San Francisco Comprehensive Cancer Center, 1600 Divisadero Street, Box 1702, San Francisco, CA 94143, USA; 2 Department of Obstetrics and Gynecology, Division of Gynecologic Oncology, Stanford University School of Medicine, Stanford Cancer Center, 875 Blake Wilbur Drive, MC 5827, Stanford, CA 94305, USA; 3 Department of Medicine, Division of Hematology and Oncology, Chao Family Comprehensive Cancer Center, University of California, Irvine – Medical Center, 101 City Drive, Orange, CA 92868, USA; 4 Department of Radiation Oncology, Division of Radiation Therapy, Stanford University School of Medicine, Stanford Cancer Center, 875 Blake Wilbur Drive, MC 5827, Stanford, CA 94305, USA

**Keywords:** lymph node resection, ovarian cancer, survival

## Abstract

The aim of the study is to determine the role of lymphadenectomy in advanced epithelial ovarian cancer. The data were obtained from the Surveillance, Epidemiology and End Results (SEER) program reported between 1988 and 2001. Kaplan–Meier estimates and Cox proportional hazards regression models were used for analysis. Of 13 918 women with stage III–IV epithelial ovarian cancer (median age: 64 years), 87.9% were Caucasian, 5.6% African Americans, and 4.4% Asians. A total of 4260 (30.6%) underwent lymph node dissections with a median number of six nodes reported. For all patients, a more extensive lymph node dissection (0, 1, 2–5, 6–10, 11–20, and >20 nodes) was associated with an improved 5-year disease-specific survival of 26.1, 35.2, 42.6, 48.4, 47.5, and 47.8%, respectively (*P*<0.001). Of the stage IIIC patients with nodal metastases, the extent of nodal resection (1, 2–5, 6–10, 11–20, and >20 nodes) was associated with improved survivals of 36.9, 45.0, 47.8, 48.7, and 51.1%, respectively (*P*=0.023). On multivariate analysis, the extent of lymph node dissection and number of positive nodes were significant independent prognosticators after adjusting for age, year at diagnosis, stage, and grade of disease. The extent of lymphadenectomy is associated with an improved disease-specific survival of women with advanced epithelial ovarian cancer.

Ovarian cancer is the most lethal gynaecologic malignancy, and is the fifth-highest cause of cancer deaths in women in the United States. In 2006, it was estimated that 20 180 women will be diagnosed with ovarian cancer, and 15 310 will die of the disease (Jemal *et al*, 2006). The standard of care in the treatment of patients with advanced-stage epithelial ovarian cancer includes primary cytoreduction surgery, followed by a platinum-based chemotherapy regimen (Marsden *et al*, 2000; Bristow *et al*, 2002).

A major controversy in the surgical treatment of advanced-stage ovarian cancer concerns the optimal management of the retroperitoneal lymph nodes. Approaches ranging from biopsy of only grossly enlarged nodes to systematic dissection of bilateral pelvic and paraaortic lymph nodes have been employed. Retroperitoneal lymph node involvement is reported in 50–75% of patients with advanced-stage disease at the time of primary surgery (Chen and Lee, 1983; Burghardt *et al*, 1989, 1991; Onda *et al*, 1996). It is unclear whether lymphadenectomy aids in better staging of patients, or whether the procedure itself has therapeutic value by debulking gross and occult disease. Prior retrospective reports support the role of lymphadenectomy in ovarian epithelial cancer (Burghardt *et al*, 1986; Scarabelli *et al*, 1995). However, others have not found a benefit associated with systematic lymphadenectomy (Spirtos *et al*, 1995).

Benedetti Panici *et al* (2005) reported the results of a prospective trial on 427 patients with optimally debulked stage IIIB–IV epithelial ovarian cancer randomised to systematic pelvic and paraaortic lymphadenectomy *vs* resection of bulky nodes only. Those who underwent a systematic lymph node dissection had a 7-month improvement in progression-free survival (29.4 *vs* 22.4 months). However, they were unable to demonstrate a significant overall survival benefit.

Given that most of the prior retrospective studies have been limited by a small sample size, we performed a large population-based study to investigate the role of lymphadenectomy in patients with advanced-stage ovarian cancers. Our study analysed the disease-specific survival outcomes of 13 918 patients diagnosed with advanced-stage epithelial ovarian cancer to determine the potential role of lymph node dissection.

## MATERIALS AND METHODS

Demographic, clinicopathologic, surgical, and survival information of women diagnosed with epithelial ovarian cancer during the period from 1 January 1988 to 31 December 2001 were extracted with permission from the Surveillance, Epidemiology and End Results (SEER) program of the United States National Cancer Institute. This data represent approximately 14% of the US population and are reported from 12 population-based registries including San Francisco-Oakland, Connecticut, metropolitan Detroit, Hawaii, Iowa, New Mexico, Seattle (Puget Sound), Utah, metropolitan Atlanta, Alaska, San Jose-Monterey, and Los Angeles (Hankey *et al*, 1999).

Only patients with advanced (stage III–IV) disease who had undergone a surgical staging procedure were included in the analysis. Patients with borderline tumours of the ovary, as well as those patients with germ cell, sex cord stromal, and sarcoma histologies, were excluded. Patients were assigned to the following categories based on race classifications described by the SEER program: Caucasian, African Americans, Asians, and others. Asians were defined as Chinese, Japanese, Korean, Vietnamese, and Filipino. All other race and ethnicity groups were defined as others.

In the assessment of demographic trends in the patient cohort, and to determine 5-year disease-specific survival, *χ*^2^-tests and Kaplan–Meier analyses with log-rank tests were performed. The outcome of specific interest was death due to ovarian cancer (disease-specific survival). The Cox proportional hazards model was used to investigate the significance of extent of lymph node resection (characterised by the number of lymph nodes reported) after adjusting for other patient features, including age, race, year of diagnosis, stage, and grade, and number of positive nodes. For the analysis of the patients who had received a lymph node dissection as part of their primary surgical treatment, the patients were grouped based on the extent of the lymphadenectomy (1, 2–5, 6–10, 11–20, and >20 nodes) to meet proportionality assumptions. All data were analysed using Intercooled STATA (version 8.0; STATA Corporation, College Station, TX, USA) and SAS (version 6.12; SAS Inc., Cary, NC, USA).

## RESULTS

Of the 13 918 patients with stage III–IV epithelial ovarian cancer, the median age was 62.7 years (range: 12–101). The median year of diagnosis was 1995. The majority of patients were Caucasian (87.9%), with African Americans, Asians, and others making up 5.6, 4.4, and 1.9% of patients, respectively (Table 1). After undergoing surgical staging, 8062 patients had stage III disease with 448 having stage IIIA, 672 stage IIIB, 4576 stage IIIC, and 5856 patients having stage IV ovarian cancer. Of the entire cohort, 4260 had a lymphadenectomy performed as part of their surgery, with a median number of 6 nodes removed (range: 1–90). For patients with positive nodes, the median number of positive nodes was two (range: 1–54) and the median number of total nodes recovered was seven (range: 1–90). In the study group, 66.8% of tumours were of serous histology, 9.2% endometrioid, 5.6% mucinous, and 2.8% clear cell. A large proportion of patients had grade 3 disease (60%), with 4.2 and 17.6% of patients having grade 1 and 2 disease, respectively (Table 2). The median time of follow-up was 22 months (range: 0–167 months).

**Figure 1:**
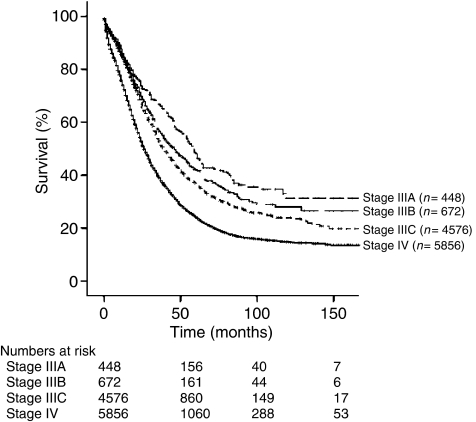
Kaplan–Meier analysis based on stage of disease (*n*=13 918; *P*<0.001).

The 5-year disease-specific survival for those ⩽64 years was 37.1 *vs* 24.4% for those >64 years (*P*<0.001). The survival of patients with stage IIIA was 48.0%, IIIB 42.1%, and IIIC 36.7%. Stage IV patients had a survival of 24.1% (Figure 1). Women with grade 1, 2, and 3 tumours had survivals of 56.9, 33.4, and 29.2%, respectively (*P*<0.001). The survival estimates based on histologies were serous 30.6%, endometrioid 43.6%, mucinous 33.3%, and clear cell 25.5% (*P*<0.001).

**Figure 2:**
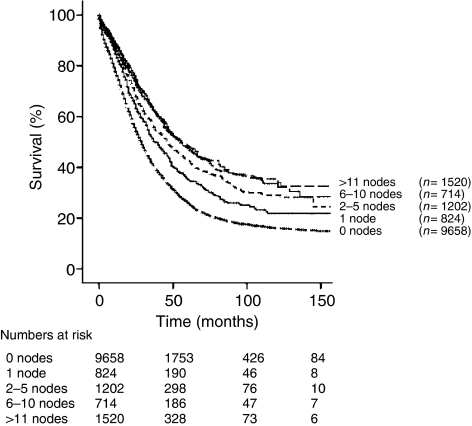
Kaplan–Meier analysis of patients by extent of lymphadenectomy (*n*=13 918; *P*<0.001).

To evaluate the effect of the extent of lymph node dissection, our study cohort was divided into six groups: patients who had 0, 1, 2–5, 6–10, 11–20, and >20 nodes reported. For all stages, we found that the removal of increasing numbers of lymph nodes was associated with a significant increase in 5-year disease-specific survival (Table 3). The findings of 0, 1, 2–5, 6–10, 11–20, and >20 lymph nodes were associated with survivals of 26.1, 35.2, 42.6, 48.4, 47.5, and 47.8%, respectively (*P*<0.001; Figure 2 also). The effect of extent of lymphadenectomy by histological subtype and by grade is shown in Table 3. The 5-year disease-specific survival rates were found to significantly increase when more nodes were resected, within all grades and histologic types. In an analysis of those patients (*n*=2563) who were found to have nodal metastases, the removal of a total of 1, 2–5, 6–10, 11–20, and >20 lymph nodes was associated with survival rates of 32.8, 36.8, 38.7, 42.0, and 41.7%, respectively (*P*=0.002; Table 4). For 2188 patients with stage IIIC disease and positive lymph nodes, survival was noted to be significantly associated with a more extensive lymphadenectomy. Additionally, the effect of the number of positive nodes was investigated in subgroups of patients with IIIC-IV node-positive disease, dividing patients into those with 1, 2–5, and >5 positive lymph nodes. When 1, 2–5, and >5 positive lymph nodes were found, the removal of increasing numbers of negative lymph nodes was associated with improved survival (Table 4).

On multivariate analysis, the extent of lymph node dissection, both as a categorical and continuous variable, persisted as an independent prognostic factor. In addition, age at diagnosis, stage, grade, histologic cell type, number of positive nodes, and year of diagnosis were also found to be significant prognosticators (Table 5).

## DISCUSSION

The standard therapy for advanced epithelial ovarian carcinoma includes total hysterectomy, bilateral salpingo-oophorectomy, omentectomy, washings, blind biopsies of diaphragm and peritoneum, and optimal surgical cytoreduction, followed by platinum-based chemotherapy. The prognostic value of complete tumour debulking on the overall survival has been demonstrated in many retrospective analyses (Piver *et al*, 1988; Covens, 2000; Bristow *et al*, 2002). On the basis of a recent meta-analysis of 81 cohorts of patients with stage III–IV disease, it was found that for each 10% increase in maximal cytoreduction, there was an associated 5.5% increase in median survival (Bristow *et al*, 2002). However, the role of retroperitoneal nodal resection remains unclear, particularly for advanced-stage disease.

In a retrospective review of 127 patients, Carnino *et al* (1997) reported that the probability of finding a lymph node metastasis was significantly higher when more lymph nodes were removed. These authors suggested that systematic lymphadenectomy should be performed, rather than lymph node sampling, to determine the therapeutic impact of lymph node resection in epithelial ovarian cancers. Determining nodal metastases by palpation at the time of surgery has been found to have significant limitations (Petru *et al*, 1994; Arango *et al*, 2000; Eisenkop and Spirtos, 2001; Tangjitgamol *et al*, 2003).

The potential benefit of performing of a systematic lymphadenectomy in the primary surgical evaluation of presumed early-stage ovarian cancer patients has been previously investigated. The value of systematic retroperitoneal node dissection may be associated with the upstaging of patients with clinical stage I cancers, which directs them to further treatment with chemotherapy. Furthermore, when initial surgical staging is adequate, patients with low-risk disease may be spared cytotoxic chemotherapy (Trimbos *et al*, 2003; Chan *et al*, 2007).

The role of systematic lymphadenectomy in advanced stages of ovarian cancer is somewhat unclear. Some prior studies have found an association between systematic node dissection and improved survival. In a retrospective study of 82 patients with stage III disease, Burghardt *et al* (1986) showed that pelvic lymphadenectomy was associated with an improved survival compared with those patients who did not have a lymphadenectomy. In a retrospective review of 150 epithelial cancer patients based on the Tokai Ovarian Tumor Study Group, Kikkawa *et al* (1995) found that the performance of a lymphadenectomy was associated with improved survival in a multivariate analysis after controlling for the effects of stage, residual disease, and histological subtype (Hazard Ratio: 0.677; *P*=0.0497).

In a randomised, controlled multi-institutional study of 427 advanced-staged optimally debulked patients, Benedetti Panici *et al* (2005) showed a 7-month improvement in disease-free survival in those who underwent a systematic lymphadenectomy compared with patients who had removal of only pathologically enlarged lymph nodes. In another randomised trial of 268 patients with epithelial ovarian cancer macroscopically confined to the pelvis after cytoreductive surgery, Maggioni *et al* (2006) compared the effects of a systematic lymphadenectomy to random sampling of retroperitoneal lymph nodes. These authors revealed that systematic lymphadenectomy was associated with an improvement in both progression-free and overall survival; however, neither was statistically significant. The investigators stated that this trial lacked the power to detect a significant difference between the two groups. These two studies may also have been limited by the short follow-up duration for assessing long-term survival outcomes (Benedetti Panici *et al*, 2005). As such, we performed a large population-based study to evaluate the potential role of an extensive lymphadenectomy in women diagnosed with advanced-stage epithelial ovarian cancer.

In this report of 13 918 women with stages III–IV ovarian cancer, 4260 patients had a dissection of at least one lymph node performed as part of their initial surgical evaluation. Our data suggested that a more extensive lymph node dissection was associated with an improved 5-year disease-specific survival. These findings were consistent in patients within substages of stage III disease and those with nodal metastases. Although an increase in the number of positive nodes was associated with a worsened survival, the removal of 1, 2–5, 6–10, and 11–20 nodes improved the outcomes of these patients from 32.8, 36.8, 38.7, 42.0, and 41.7%, respectively. More importantly, multivariate analysis demonstrated that a more extensive node resection, both as a categorical and continuous variable, was associated with an improved survival after adjusting for age, stage, grade, number of positive nodes, and year of diagnosis (Table 5).

This study is one of the largest series to evaluate the role of lymphadenectomy in surgically staged advanced ovarian cancer patients. A large proportion of these patients had an extensive lymph node resection; in fact, 707 patients had >20 lymph nodes removed. Given the large size of this cohort, with 13 918 patients, we were able to perform subset analyses on node-positive stage IIIC and/or IV patients showing consistent results. Similar to the results of a randomised trial, our data also showed that metastatic lymph node involvement is associated with poorer survival. However, in our current separate analysis of over 2563 patients with stage IIIC disease and nodal metastases, we were able to perform a detailed subset analysis showing that increasing numbers of metastatic lymph nodes (1, 2–5, and >5) is associated with a worsened survival (40.1, 37.0, and 35.6%, respectively).

Our analysis was limited by the lack of information on surgeon's subspecialty, volume of residual disease, medical comorbidities, location of nodal resection (pelvic *vs* paraaortic), adjuvant chemotherapy, and treatment of recurrence. In particular, the extent of extranodal residual disease in stage IIIC and IV patients and its potential impact on the extent of lymphadenectomy were not available in the SEER database. Nevertheless, even among those with stage IIIA disease, defined as microscopic disease in the upper abdomen, the extent of nodal dissection (6–10, 11–20, and >20 nodes) was associated with an improved survival from 61.5, 71.4, and 74.7%, respectively. Moreover, there was no central pathology review. Patients who had a less extensive lymphadenectomy may have had significant medical and/or surgical comorbidities, thus, representing patients with poor prognostic cancers. Furthermore, owing to the retrospective nature of this analysis, there may exist a selection bias where those patients who underwent a more extensive lymphadenectomy may have had less comorbidity, as well as having tumours with more favourable prognostic features. In addition, the extent of a lymphadenectomy may not be truly reflected by the reported number of recovered nodes in our study. Clearly, the extent of the nodal resection by the surgeon as well as comprehensive processing of the specimens by the pathologists influences nodal recovery. In addition, a more thorough lymphadenectomy may be a marker for quality comprehensive medical and surgical care rather than the procedure itself resulting in the improved survival of these patients. Lastly, there are certain patients in whom lymph node sampling or lymphadenectomy may not be feasible owing to comorbidity factors, blood loss, or body habitus. In a prospective randomised trial reported by Benedetti Panici *et al* (2005) women who underwent a systematic lymphadenectomy were found to have more postoperative complications, mostly consisting of lymphocytes or lymphoedema. Furthermore, the median operating time was 90 min longer and blood loss was 350 ml higher, with 12% more blood transfusions given when a systematic lymphadenectomy was performed.

There are several possible mechanisms that may explain the improvement in survival that was found to be associated with a more extensive lymphadenectomy in advanced cancers. A more complete lymphadenectomy is likely to remove occult microscopic disease, resulting in a more complete cytoreduction. In the randomised trial reported by Benedetti Panici *et al* (2005), patients with stage IIIB–C and IV epithelial ovarian cancer randomised to undergo systematic pelvic and paraaortic lymphadenectomy were found to have a statistically significant increase in positive lymph nodes compared to those randomised to resection of bulky nodes only (70 *vs* 42%; *P*<0.001). Thus, compared to those who had a limited lymphadenectomy, 28% more patients in the extensive lymphadenectomy arm benefited from cytoreduction of occult nodal metastases. A meta-analysis of the survival effect of maximum cytoreductive surgery in advanced ovarian carcinoma reported that each 10% increase in maximum cytoreduction was associated with a 5.5% increase in median survival time (Bristow *et al*, 2002). The magnitude of improved survival reported in our current study is consistent with these estimates, suggesting that the improvement in disease-specific survival may be associated with the removal of additional occult disease.

Furthermore, an extensive lymph node resection may lead to an improvement in survival by removing micrometastatic disease within the lymph nodes that may be resistant to chemotherapy. Prior studies on patients who underwent chemotherapy followed by second-look surgery showed that 33.3–65.3% of patients with advanced-stage disease had residual disease in the retroperitoneal lymph nodes (Burghardt and Winter, 1989; Baiocchi *et al*, 1998). These studies suggested that chemotherapy appears to have minimal effect on tumour deposit in the nodes; thus, retroperitoneal lymphadenectomy should be an integral component of ovarian cancer cytoreductive surgery.

In summary, our retrospective analysis suggests that the extent of lymphadenectomy is associated with an improvement in disease-specific survival in patients with advanced ovarian carcinoma. Furthermore, the extent of nodal disease provides additional prognostic information. Further trials are warranted to investigate the treatment of these high-risk patients with nodal metastases.

## Figures and Tables

**Table 1:** Patient characteristics

	**Total (*n*=13 918) No. (%)^a^**	**0 nodes (*n*=9658) No. (%)^a^**	**1 node (*n*=824) No. (%)^a^**	**2–5 nodes (*n*=1202) No. (%)^a^**	**6–10 nodes (*n*=714) No. (%)^a^**	**11–20 nodes (*n*=813) No. (%)^a^**	**>20 nodes (*n*=707) No. (%)^a^**
*Age at diagnosis (years)*							
Mean	62.7 (±0.1)	64.1 (±0.1)	60.9 (±0.4)	59.9 (±0.4)	59.1 (±0.5)	58.1 (±0.5)	57.1 (±0.5)
Median (range)	64.0 (12–101)	65.0 (13–101)	61.0 (22–90)	60.0 (20–94)	59.5 (20–93)	58.0 (12–90)	57.0 (15–91)
							
Median year of diagnosis	1995	1994	1995	1996	1997	1997	1998
							
*Race*							
Caucasians	12240 (87.9%)	8498 (88.0%)	709 (86.0%)	1037 (86.3%)	629 (88.1%)	741 (91.1%)	626 (88.5%)
African Americans	782 (5.6%)	549 (5.7%)	57 (6.9%)	83 (6.9%)	41 (5.7%)	32 (3.9%)	20 (2.8%)
Asians^b^	612 (4.4%)	407 (4.2%)	36 (4.4%)	57 (4.7%)	30 (4.2%)	31 (3.8%)	51 (7.2%)
Others^c^	267 (1.9%)	195 (2.0%)	22 (2.7%)	24 (2.0%)	12 (1.7%)	8 (1.0%)	6 (0.8%)
Unknown	17 (0.1%)	9 (0.1%)	0 (0.0%)	1 (0.1%)	2 (0.3%)	1 (0.1%)	4 (0.6%)
							
*Year of diagnosis*							
1988–1992	4142 (29.8%)	3288 (34.0%)	265 (32.2%)	267 (22.2%)	129 (18.1%)	122 (15.0%)	71 (10.0%)
1993–1997	5327 (38.3%)	3766 (39.0%)	283 (34.3%)	489 (40.7%)	276 (38.7%)	296 (36.4%)	217 (30.7%)
1998–2001	4449 (31.9%)	2604 (27.0%)	276 (33.5%)	446 (37.1%)	309 (43.3%)	395 (48.6%)	419 (59.3%)

a Percentage of patients for given parameter.

b Asians were defined as Chinese, Japanese, Korean, Vietnamese, and Filipino.

c Others were defined as all other race/ethnicity parameters.

**Table 2:** Clinicopathologic data

	**Total (*n*=13 918) No. (%)^a^**	**0 nodes (*n*=9658) No. (%)^a^**	**1 node (*n*=824) No. (%)^a^**	**2–5 nodes (*n*=1,202) No. (%)^a^**	**6–10 nodes (*n*=714) No. (%)^a^**	**11–20 nodes (*n*=813) No. (%)^a^**	**>20 Nodes (*n*=707) No. (%)^a^**	**Chi-square Test *P*-value**
*Stage of disease*								
Stage IIIA	448 (3.2)	281 (2.9)	25 (3.0)	56 (4.7)	36 (5.0)	33 (4.1)	17 (2.4)	*P*<*0.001*
Stage IIIB	672 (4.8)	477 (4.9)	26 (3.2)	58 (4.8)	42 (5.9)	46 (5.7)	23 (3.3)	
Stage IIIC	4576 (32.9)	2384 (24.7)	415 (50.4)	592 (49.3)	350 (49.0)	429 (52.8)	406 (57.4)	
Stage III, NOS	2366 (17.0)	2010 (20.8)	62 (7.5)	107 (8.9)	58 (8.1)	73 (9.0)	56 (7.9)	
Stage IV	5856 (42.1)	4506 (46.7)	296 (35.9)	389 (32.4)	228 (31.9)	232 (28.5)	205 (29.0)	
								
*Grade of disease*								
Grade 1	580 (4.2)	366 (3.8)	30 (3.6)	47 (3.9)	43 (6.0)	47 (5.8)	47 (6.6)	*P*=0.005
Grade 2	2443 (17.6)	1684 (17.4)	135 (16.4)	211 (17.6)	123 (17.2)	164 (20.2)	126 (17.8)	
Grade 3	8349 (60.0)	5673 (58.7)	531 (64.4)	765 (63.6)	437 (61.2)	490 (60.3)	453 (64.1)	
Unknown	2546 (18.3)	1935 (20.0)	128 (15.5)	179 (14.9)	111 (15.5)	112 (13.8)	81 (11.5)	
								
*Histology*								
Serous	9294 (66.8)	6474 (67.0)	553 (67.1)	805 (67.0)	457 (64.0)	532 (65.4)	473 (66.9)	*P*<0.001
Endometrioid	1275 (9.2)	778 (8.1)	78 (9.5)	137 (11.4)	91 (12.7)	100 (12.3)	91 (12.9)	
Mucinous	775 (5.6)	554 (5.7)	46 (5.6)	61 (5.1)	36 (5.0)	39 (4.8)	39 (5.5)	
Clear cell	394 (2.8)	206 (2.1)	23 (2.8)	62 (5.2)	35 (4.9)	39 (4.8)	29 (4.1)	
Others or NOS	2180 (15.7)	1646 (17.0)	124 (15.0)	137 (11.4)	95 (13.3)	103 (12.7)	75 (10.6)	

a Percentage of patients for given parameter.

**Table 3:** Five-year disease-specific survival based on the extent of lymphadenectomy and clinicopathologic characteristics

	**No.**	**Total % (s.e.)**	**0 nodes % (s.e.)**	**1 node % (s.e.)**	**2–5 nodes % (s.e.)**	**6–10 nodes % (s.e.)**	**11–20 nodes % (s.e.)**	**>20 nodes % (s.e.)**	**Log-rank**
*Stage of disease*									*P*<0.001
Stage III–IV	13 918	31.1 (0.5)	26.1 (0.5)	35.2 (2.0)	42.6 (1.8)	48.4 (2.4)	47.5 (2.3)	47.8 (2.8)	*P*<0.001
Stage III	8062	36.7 (0.7)	30.5 (0.8)	37.4 (2.7)	47.7 (2.2)	55.2 (2.9)	51.6 (2.8)	54.5 (3.2)	*P*<0.001
Stage IIIA	448	48.0 (2.8)	40.4 (3.3)	33.9 (12.6)	66.8 (8.4)	61.5 (10.5)	71.4 (9.6)	74.7 (17.5)	*P*<0.001
Stage IIIB	672	42.1 (2.4)	35.1 (2.7)	41.0 (12.9)	55.9 (8.0)	74.0 (7.6)	61.0 (8.8)	81.1 (10.1)	*P*=0.001
Stage IIIC	4576	36.7 (0.9)	29.0 (1.2)	36.9 (3.1)	45.0 (2.6)	47.8 (3.6)	48.7 (3.3)	51.1 (3.5)	*P*<0.001
Stage IV	5856	24.1 (0.7)	21.4 (0.7)	31.3 (3.1)	33.1 (2.9)	34.6 (4.0)	38.3 (4.1)	32.2 (5.0)	*P*<0.001
									
*Grade of disease*									*P*<0.001
Grade 1	580	56.9 (2.4)	49.3 (3.0)	44.5 (11.2)	67.3 (7.7)	71.9 (7.8)	75.2 (7.8)	77.3 (7.6)	*P*<0.001
Grade 2	2443	33.4 (1.2)	28.0 (1.3)	37.3 (5.6)	45.0 (4.3)	59.1 (5.6)	49.4 (5.1)	48.3 (6.1)	*P*<0.001
Grade 3	8349	29.2 (0.6)	24.2 (0.7)	32.6 (2.5)	40.4 (2.3)	44.6 (3.1)	43.5 (2.9)	46.4 (3.6)	*P*<0.001
									
*Histology*									*P*<0.001
Serous	9294	30.6 (0.6)	26.1 (0.7)	32.2 (2.5)	41.6 (2.2)	49.7 (3.0)	45.6 (2.9)	44.0 (3.5)	*P*<0.001
Endometrioid	1275	43.6 (1.6)	35.5 (2.0)	53.1 (6.4)	49.8 (5.0)	50.7 (6.7)	63.0 (5.9)	75.4 (5.6)	*P*<0.001
Mucinous	775	33.3 (2.0)	28.1 (2.2)	41.6 (8.5)	48.7 (8.1)	46.1 (10.1)	51.3 (9.6)	47.0 (9.7)	*P*<0.001
Clear cell	394	25.5 (2.9)	18.3 (3.6)	10.1 (9.1)	38.0 (7.0)	39.9 (10.4)	34.4 (9.4)	37.6 (10.3)	*P*=0.007

s.e.=standard error.

**Table 4:** Five-year disease-specific survival analysis for node-positive stage IIIC–IV patients based on the extent of lymphadenectomy and number of positive nodes

			**Total number of nodes removed**					
**Positive node number**	**No.**	**Total % (s.e.)**	**1 node % (s.e.)**	**2–5 nodes % (s.e.)**	**6–10 nodes % (s.e.)**	**11–20 nodes % (s.e.)**	**>20 nodes % (s.e.)**	**Log-rank**
All patients	2563	38.0 (1.3)	32.8 (2.6)	36.8 (2.4)	38.7 (3.1)	42.0 (2.9)	41.7 (3.3)	*P*=0.002
1 positive node	1067	40.1 (1.9)	32.8 (2.6)	45.8 (4.1)	48.1 (6.1)	43.7 (6.5)	58.2 (8.1)	*P*<0.001
2–5 positive nodes	972	37.0 (2.1)	—	31.4 (3.0)	38.9 (4.5)	44.2 (4.7)	40.2 (5.7)	*P*<0.001
>5 positive nodes	524	35.6 (2.8)	—	—	29.6 (5.5)	38.7 (4.6)	36.5 (4.6)	*P*=0.883

s.e.=standard error.

**Table 5:** Multivariate analysis

**Prognostic factor**	**Hazard ratio**	**95% confidence interval**	***P*-value**
Age at diagnosis^a^	1.018	1.016–1.019	*P*<0.005
			
Year of diagnosis^b^	0.977	0.970–0.984	*P*<0.005
			
Stage^c^	1.266	1.220–1.315	*P*<0.005
			
Grade^d^	1.933	1.684–2.219	*P*<0.005
			
Histology^e^	1.994	1.716–2.316	*P*<0.005
			
Extent of lymphadenectomy^f^	0.911	0.861–0.964	*P*=0.001
			
Positive nodes^g^	1.338	1.215–1.473	*P*<0.005

a Continous.

b Continous.

c Stage IIIA/B *vs* IIIC *vs* IV.

d Grade 1 *vs* 2–3.

e Others *vs* clear cell.

f 0 *vs* 1 *vs* 2–5 *vs* 6–10 *vs* ⩾11.

g No *vs* yes.
